# “Holding–Cuddling” and Sucrose for Pain Relief During Venepuncture in Newborn Infants: A Randomized, Controlled Trial (CÂSA)

**DOI:** 10.3389/fped.2020.607900

**Published:** 2021-01-14

**Authors:** Karole Hoarau, Marie Line Payet, Laurence Zamidio, Francesco Bonsante, Silvia Iacobelli

**Affiliations:** ^1^Néonatologie, Réanimation Néonatale et Pédiatrique, CHU, Saint Pierre, France; ^2^Centre d'Etudes Périnatales de l'Océan Indien (EA7388), Université de la Réunion, Saint Pierre, France

**Keywords:** child-nursing, suction, dextrose, APN score, intensive care

## Abstract

**Objectives:** Oral sucrose is commonly used to provide analgesia to neonates during painful procedures, such as venepuncture. The additional benefits of reducing pain during venepuncture when oral sucrose is combined with nonpharmacological strategies have not been extensively studied. This randomized controlled trial compared the efficacy of oral sucrose with nonnutritive sucking vs. oral sucrose with nonnutritive sucking plus “holding–cuddling” for pain management during venepuncture in term infants from birth to 3 months of life.

**Methods:** Seventy-eight infants were equally randomized to receive 24% oral sucrose with nonnutritive sucking (control group) or 24% oral sucrose with nonnutritive sucking plus “holding–cuddling” (being held in a secure, cuddling position; experimental group) before venepuncture. Behavioral response to pain was measured by the 0–10 ranking scale “acute pain for neonates (APN)” at 30 and 60 s after venepuncture.

**Results:** Within the study sample, APN scores were ≥ 2 for 32/68 (47%) infants. “Holding–cuddling” did not significantly reduce mean APN scores at 30 and 60 s, but the rate of infants experiencing a high pain score (APN ≥ 8) at 60 s after the venepuncture was significantly lower in the experimental group compared to controls [4/34 vs. 12/34 (*p* = 0.04)].

**Conclusions:** Venepuncture is a painful procedure in newborn and young infants. The implementation of behavioral strategies in association with oral sucrose may mitigate pain during this procedure.

**Clinical Trial Registration:** This trial was registered at http://clinicaltrials.gov/ (NCT number 02803723).

## Introduction

Every day, worldwide, most newborn babies and young infants undergo painful procedures. The pain-reducing properties of oral sweet solutions for skin breaking procedures have been extensively demonstrated in systematic reviews and meta-analyses (1, 2). However, several questions remain (3), and among the critical issues raised in the conclusion of the last Cochrane review (1), one of them is to address the effects of sucrose in combination with a nonpharmacological intervention for acute pain.

Since one of the first trials demonstrated the analgesic effects of concentrated sucrose solutions in full-term infants (4), a relevant comment in the literature has addressed the interest of minimizing the discomfort of the painful procedure by asking the parent (or a member of the staff) to cuddle and soothe the baby (5). Several studies have shown the benefits of behavioral and environmental interventions for reducing pain, including the following: skin-to-skin contact (6), breastfeeding (7), acupressure, and facilitated tucking—holding the infant's arms and legs in flexed positions close to the midline of the torso (8). Most of these studies have been performed in preterm or early term newborn babies (8). An update on the current state of evidence has shown that, in full-term infants, direct breastfeeding is more effective than maternal holding, maternal skin-to-skin contact, topical anesthetics, and music therapy and is as or more effective than sweet-tasting solutions (7). In some contexts, and when breastfeeding is not possible, parental presence with adjunctive provision of skin-to-skin care or nonnutritive sucking is recommended during painful procedures (9). To date, a few randomized controlled trials have studied the beneficial effects of sucrose (with or without nonnutritive sucking) in combination with nonpharmacological strategies. Swaddling (10), facilitated tucking (11, 12), and skin-to-skin care (13) have been associated with oral sucrose to reduce pain, especially in preterm, low-birth weight infants and during transition to postnatal life (14). In our unit, according to national guidelines (15), the administration of oral sucrose with nonnutritive sucking is recommended during painful procedures in infants <4 months old. In this context, we aimed to investigate, in term neonates and young infants, the effect of oral sucrose in combination with the “holding–cuddling” on venepuncture-related pain. The aim of the study was to compare the efficacy of oral sucrose vs. oral sucrose + “holding–cuddling” as methods for pain management during venepuncture in infants under three months old.

## Patients and Methods

This was a single-center, prospective, randomized, unblinded, parallel-group, controlled trial.

### Setting and Sample

Infants were recruited in the level 3 neonatal and pediatric intensive care unit (NICU and PICU) of Saint Pierre, which is a university teaching hospital in France, from August 2016 to June 2018. Using sealed, opaque, and sequentially numbered envelopes, patients were randomly assigned to receive 24% oral sucrose (control group) with nonnutritive sucking or 24% oral sucrose with nonnutritive sucking + “holding–cuddling” (experimental group). We calculated that, in order to achieve 80% power to show in the experimental group a 2.03 points reduction in the acute pain scale for neonates (APN) (16) at 30 s after venepuncture, 34 infants would be required in each group (standard deviation equal to 2.67 in both groups). The random allocation sequence was generated by the methodology center; participants were enrolled by investigator nurses and assigned to intervention according to randomization.

Infants who required a venepuncture for blood sample were recruited if they had the following two criteria: born at term (gestational age at birth ≥37 weeks of gestation) and age under 3 months of life. Non-inclusion criteria were as follows: invasive mechanical ventilation, intravenous or oral sedation or analgesia, contraindication to oral sucrose, or severe neurodevelopmental disability.

### Interventions

After randomization, each infant allocated in the control group received, 2 min before the venepuncture, 2 mL of 24% oral sucrose (BABICALMINE S®) administered into their mouth by a pipette and a pacifier. Infants in the control group were not held during venepuncture, and the procedure was performed with the infant positioned in hospital cradle.

**Figure 1:**
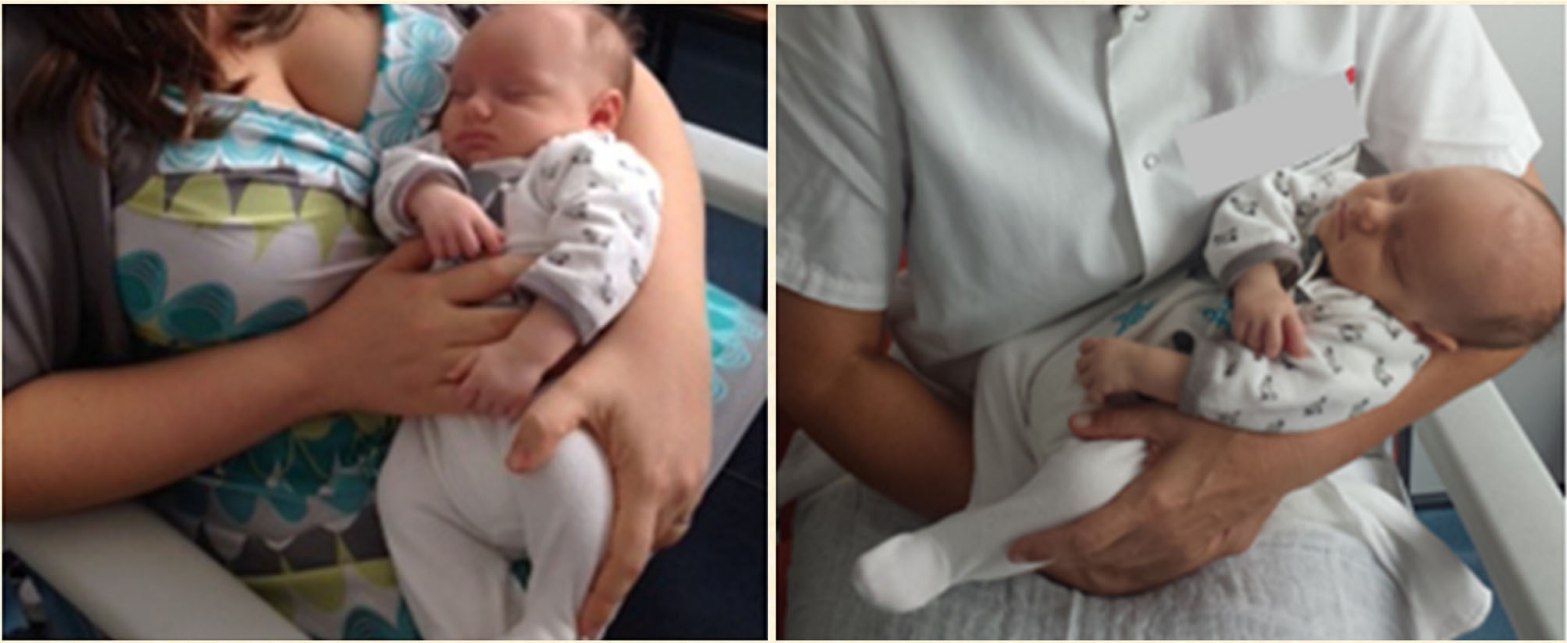
The holding–cuddling as performed by the mother or by the childcare assistant.

Each infant allocated in the experimental group was placed, 5 min before the venepuncture, in the mother's or in the staff childcare assistant's hug, where the infant was held in a secure, cuddling, and soothing position— “holding–cuddling” (Figure 1); in addition, 2 min before the venepuncture, the infant was administered with 2 mL of 24% oral sucrose as described above.

Aside from the required monitoring of adverse effects for all clinical trials, there were no specific adverse effects monitored for in regards to the holding intervention.

### Data Collection

Baseline demographic and clinical characteristics of each group were collected.

During the hospitalization, arterial oxygen saturation rate (SpO_2_) and heart rate (HR) were continuously monitored. For the purpose of the research questions, the baby's baseline HR and SpO_2_ were recorded immediately before the venepuncture (time 0, t0) and 30 s after (t30).

Pain was measured by the APN score, which is a behavioral scale validated to rate acute pain in neonates (16). Three items (facial expression, limb movements, and vocal expression) are evaluated, and the score ranges from 0 (no pain) to 10 (maximum pain) (13) (Table 1). An APN score ≥ 2 identifies a painful procedure. The scale was assessed at 30 and 60 s after the venepuncture. The assessors of all variables of interest were registered pediatric staff nurses of the staff, not participating in the research project.

**Table 1:** APN behavioral scale for rating acute pain in neonates.

**Facial expressions**	
Calm	0
Snivels and alternates gentle eye opening and closing	1
**Intensity of eye squeeze, brow bulge, and nasolabial furrow:**	
Mild, intermittent with return to calm^*^	2
Moderate^†^	3
Very pronounced, continuous^‡^	4
**Limb movements**	
Calm or gentle movements	0
**Intensity of pedaling, toes spread, legs tensed and pulled up, agitation of arms, and withdrawal reaction:**	
Mild, intermittent with return to calm^*^	1
Moderate^†^	2
Very pronounced, continuous^‡^	3
**Vocal expression**	
No complaints	0
Moans briefly (for intubated child, looks anxious, or uneasy)	1
Intermittent crying (for intubated child, expression of intermittent crying)	2
Long-lasting crying, continuous howl (for intubated child, expression of continuous crying)	3

* *Present during <1/3 of observation periods*.

† *Present during 1/3 to 2/3 of observation periods*.

‡ *Present during >2/3 of observation periods*.

The primary outcome was the APN score at 30 s from the venepuncture.

Secondary outcomes were the following: APN score at 60 s, HR and SO_2_ at 30 s, ΔHR (t30–t0), and the success rate of venepuncture.

### Validity and Reliability

The main outcome measure of this trial was measured by the APN scale, which showed internal consistency of 0.88 and an interrater reliability of 91.2 (Krippendorf) in the validation study (16). This scale is easy and fast to use, and it has had extensive reliability and validity testing under controlled research conditions (17, 18). Pain assessment during procedures is systematically collected among quality indicators in our NICU, and all registered pediatric nurses are trained and experienced with the APN tool. Finally, this is one of the five scales suggested by the American Academy of Pediatrics for assessing neonatal pain (19).

### Data Analysis

Categorical variables were presented as frequencies, and continuous variables were presented as means ± standard deviations (SD). The main analysis was performed on the groups constituted by the allocation procedure (intention-to-treat analysis), and a one-sided Mann–Whitney test was used to compare the mean values of the APN scale pain at 30 s. Comparisons between nonparametric data were performed by using the Mann–Whitney test and those between parametric data by chi-squared test (or Fisher exact when appropriate). A multiple linear regression including confounding factors was planned in case of unbalanced groups in order to check the robustness of the conclusion. The level of significance was set to 5% (*p* < 0.05) for all comparisons. Analyses were performed using the MedCalc software (version 12.3.0; MedCalcSoftware's, Ostend, Belgium).

## Results

**Figure 2:**
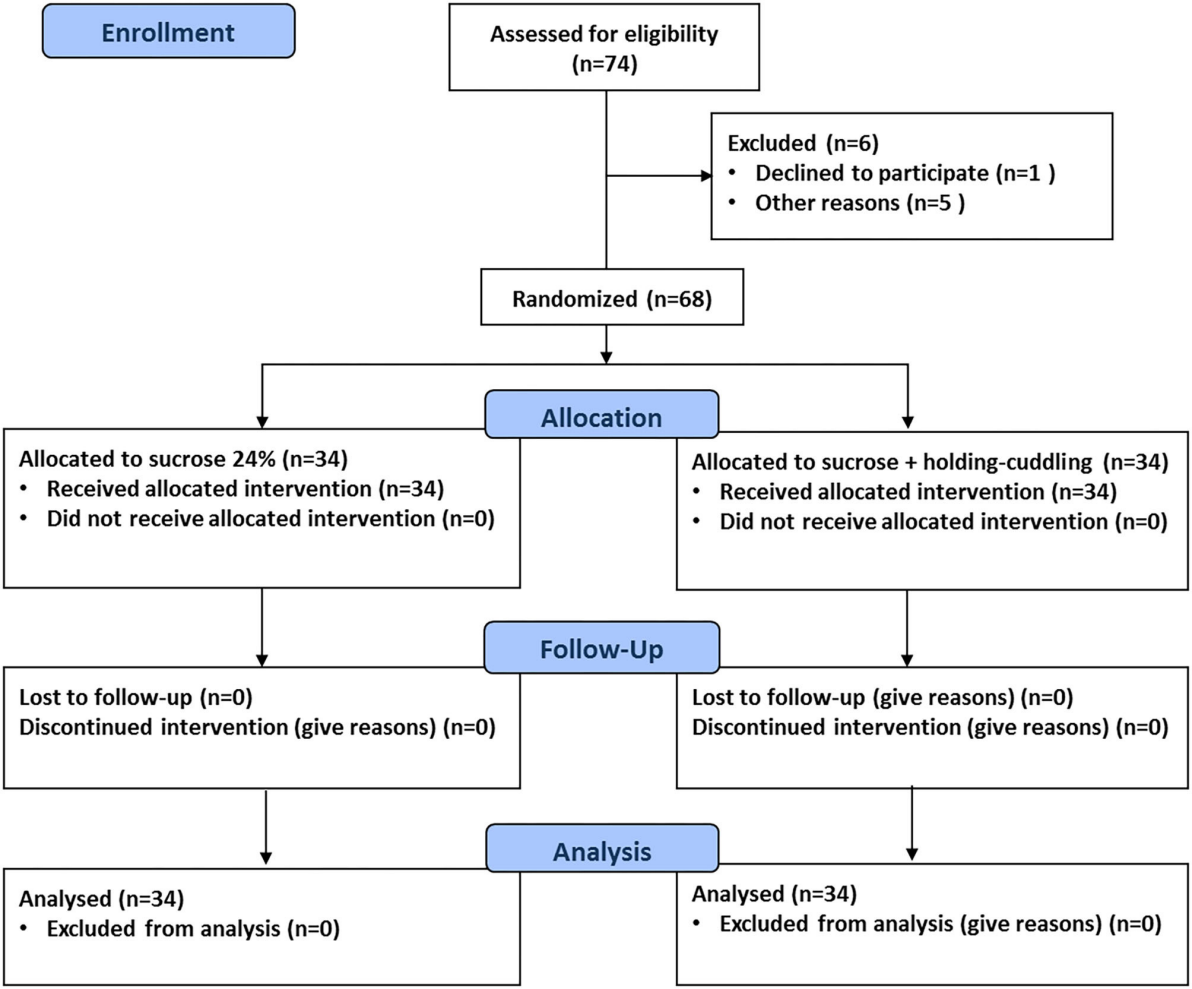
Câsa. Study flow diagram.

The flow of participants through each stage of the study is shown in Figure 2. During the study period, 68 infants in total were included, 34 in each group. There were no withdrawals after randomization with regard to intended allocation treatment, study protocol completion, and analysis of the primary outcome. There were no protocol deviations.

Overall, the study sample had a gestational age at birth of 37 ± 3.2 weeks and a birth weight of 2,897 ± 737 g; the girls to boys ratio was 30/38, and 27/68 infants were aged under 28 days of life. The mean age at the study inclusion was 17.4 ± 18.9 days of life. The most frequent reason for admission to hospital was bronchiolitis (12/68). Other reasons for admission were as follows: upper airway infections, fever, urinary tract infections, faltering growth, feeding difficulties, late onset neonatal infections, breathing difficulties, and dehydration. Baseline demographic and clinical characteristics of each group are shown in Table 2.

**Table 2:** Baseline demographic and clinical characteristics of the study population (68 infants).

**Variable**	**Control (Total = 34)**	**Experimental (Total = 34)**	***p*-value**
Gestational age at birth, weeks	37.0 ± 3.6	37.8 ± 2.7	0.34
Birth weight, grams	2,873 ± 765	2,920 ± 719	0.89
Male gender	20/34	18/34	0.81
Postnatal age <28 days	13/34	14/34	0.45
Bronchiolitis	6/34	6/34	1.00

There were no significant differences between the groups in sex, gestational age, birth weight, postnatal age, and other studied variables.

According to the APN pain scale, a total of 32 infants within the two groups (47% of the study sample) experienced a painful procedure (any APN score equal or superior to 2) during venepuncture.

The primary outcome (APN score at 30 s) was not statistically different between the two groups. Acute pain score at 60 s was lower in the experimental group than in the control group, but the difference was not statistically significant. The rate of infants experiencing a high pain score (APN ≥ 8) at 60 s after the venepuncture was significantly lower in the experimental group compared to controls. There were no significant variations in HR and SpO_2_ following venepuncture between the two groups. The site of venepuncture was the dorsal venous network of the infant hand, and the success rate of venepuncture was similar in controls and in infants with “holding–cuddling.” These results are shown in Table 3.

**Table 3:** Summary of results and outcomes for each group (68 infants).

**Variable**	**Control (*N* = 34)**	**Experimental (*N* = 34)**	***p*-value**
APN at 30 s	3.6 ± 3.7	2.8 ± 3.2	0.33
APN at 60 s	4.1 ± 4.2	2.4 ± 3.4	0.08
APN at 60 s < 2	15/34	21/34	0.22
APN at 60 s 2–4^†*^ (low)	7/34	5/34	0.11
APN at 60 s 5–7^†*^(moderate)	0/34	4/34	0.75
APN at 60 s 8–10^†*^ (high)	12/34	4/34	0.04
HR at t0 (bpm)	160 ± 19	163 ± 20	0.55
HR at t30 (bpm)	174 ± 21	172 ± 22	0.63
ΔHR (t30–t0) (bpm)	14 ± 17	9 ± 13	0.18
SpO_2_ at t0	98,0 ± 2.8	97.9 ±2.7	0.72
SpO_2_ at t30	97.4 ± 4.1	97.6 ± 3.1	0.83
Venepuncture success rate^†^	30/34	32/34	0.67

APN, acute pain scale for neonates; HR, heart rate; SpO_2_, arterial oxygen saturation rate; bpm, beats per minute. All data, excluded data with ^†^, are expressed as means ± SD. All data, excluded data with *, were prespecified in the analysis.

In the experimental group, the “holding–cuddling” was performed by the infant's mother in 16 babies and by a member of the staff in the remaining 18.

## Discussion

This study confirmed what is already known—that venepuncture is a painful procedure in newborn infants (20–22)—and demonstrated that, despite the administration of sucrose plus nonnutritive sucking according to our national recommendations, a considerable number of infants experienced significant pain following venepuncture for a blood sample, as measured by an APN score equal or superior to 2. Our findings did not confirm the research hypothesis as measured by the primary outcome because the comparison of oral sucrose vs. oral sucrose + “holding–cuddling” did not show a statistical significance in reducing pain 30 s after venepuncture. However, a positive trend in favor of the intervention “holding–cuddling” was observed in terms of a reduced pain score at 60 s, and, moreover, the rate of the high APN score at 60 s from venepuncture was significantly lower in the experimental group.

To the best of our knowledge, and according to one recent systematic review (8), no study has been conducted in order to investigate the efficacy of behavioral and/or environmental interventions in association with oral sucrose. Indeed, the RCT from Sahho and colleagues (23) has compared the effects of expressed breast milk vs. 25% dextrose for pain management during this procedure, showing the better efficacy of 25% dextrose. Similar to our work, this study emphasized that venepuncture is a painful procedure with pain scores (PIPP) as high as 11 immediately after the procedure and with high scores persisting after 5 min in both groups. The authors concluded that the use of analgesics, either glucose or breast milk, does not totally alleviate the pain. As shown by a recent update, expressed breast milk alone should not be considered an adequate intervention. On the contrary, according to research-based knowledge, there is sufficient evidence to recommend direct breastfeeding for procedural pain management in full-term infants (7). Currently, studies carried out in preterm infants show an increasing evidence of benefit of environmental interventions to mitigate biobehavioral pain response (8). Even if the mechanisms of action for these interventions in reducing pain are poorly understood, they might work as they evoke neurobehaviors ensuring the fulfillment of basic biological needs, as is the case for the kangaroo-mother care for very preterm infants and for the skin-to-skin contact immediately after birth for term infants (6, 24). The most recent Cochrane review of nonpharmacological management of infant and young child procedural pain (25) has analyzed the efficacy of each nonpharmacological intervention separately for infant age (preterm, neonate, older) and has concluded that the most established evidence was for nonnutritive sucking, swaddling/facilitated tucking, and rocking/holding. These interventions are less suitable for newborn at term and young infants, and other strategies or multimodal approaches should be considered for the latter, when breastfeeding is not possible during procedural pain. Recently, one randomized clinical trial has compared the effect of a mother's hug or massage vs. no intervention on pain due to blood sampling in neonates, finding positive effects of the mother's hug on behavioral response to pain (26). In our study, the “holding–cuddling” was systematically performed by the mother when she was present and she agreed, and otherwise by one childcare assistant of the staff. This was the case in more than 50% of the infants in the experimental group, and this can be a possible explanation for the lack of superiority compared to oral sucrose alone. In our unit, the option of parent rooming in is not always available (except for very preterm infants). Due to geographic and contextual factors (travel time to referral hospital, large and single-parent families, adolescent childbearing) it can be difficult to obtain continuous parental presence during hospitalization. We did not collect data about family presence and participation during hospital care, and we acknowledge that this is a limitation of our study, as this information would have been interesting for the interpretation of results.

Other limitations of our study are as follows: lack of blinding of intervention and outcome measures, use of APN in real time as opposed to videotaping for subsequent analysis, single assessor with no inter-assessor reliability checks, and lack of stratification for the cuddle provider (mother or care provider). Finally, in our study, infants received 2 mL of 24% oral sucrose 2 min before the venepuncture. This study design can be criticized, as some studies have shown that the analgesic effect of sucrose may not persist beyond 60–120 s in term infants (1). Moreover, one randomized controlled trial found that the minimum effective dose of 24% sucrose required to treat pain associated with a single heel lance was as low as 0.1 mL in neonates (27).

The strengths of our study are as follows: randomized controlled trial design, sufficient sample size with adequate power to detect a difference if there was one, and use of a validated pain score, which is widely used in everyday practice in our unit.

In conclusion, our study provides evidence in support of the hypothesis that behavioral and environmental interventions, associated to oral sucrose, improve the management of pain procedures in newborn and young infants. The result reported did not reach statistical significance for the prespecified outcome measure, but it was significant for reducing the rate of the high pain score in the “holding–cuddling” group.

Based on our findings, implications for practice can be proposed, as our results encourage the use of a multimodal, multidisciplinary approach to acute pain management in newborn and young infants. From a clinical point of view, this approach should contemplate local and context-specific considerations. Some implications for research can be identified from our investigation, as it supports the efficacy of behavioral interventions, even if with low evidence and lack of replication. Thus, other studies with rigorous and standardized design are needed to further investigate the implementation, in clinical practice, of interventions that involve parental presence and participation to mitigate pain during infant care procedures.

## Data Availability Statement

The raw data supporting the conclusions of this article will be made available by the authors, without undue reservation.

## Ethics Statement

The studies involving human participants were reviewed and approved by French ethics committee (Registration Number 2015/72). Written informed consent to participate in this study was provided by the participants' legal guardian/next of kin. Written informed consent was obtained from the individual(s), and minor(s)' legal guardian/next of kin, for the publication of any potentially identifiable images or data included in this article.

## Author Contributions

KH conceptualized the study, included the patients, collected the data, and wrote the paper. MP contributed substantially to the conception of the study, the patient inclusion, and data collection. LZ conceptualized the study and wrote the study protocol. FB performed the statistical analyses and participated in the interpretation of the results. SI conceptualized and designed the study, oversaw the data analysis and interpretation, and critically reviewed and revised the manuscript drafts. All authors accept responsibility for the paper as submitted. All authors read and approved the final manuscript.

## Conflict of Interest

The authors declare that the research was conducted in the absence of any commercial or financial relationships that could be construed as a potential conflict of interest.
